# Ontology-based literature mining of *E. coli* vaccine-associated gene interaction networks

**DOI:** 10.1186/s13326-017-0122-4

**Published:** 2017-03-14

**Authors:** Junguk Hur, Arzucan Özgür, Yongqun He

**Affiliations:** 1 Department of Biomedical Sciences, University of North Dakota School of Medicine and Health Sciences, Grand Forks, ND 58202 USA; 2 Department of Computer Engineering, Bogazici University, Istanbul, 34342 Turkey; 3 Department of Microbiology and Immunology, Unit for Laboratory Animal Medicine, University of Michigan Medical School, Ann Arbor, MI 48109 USA; 4 Department of Microbiology and Immunology, University of Michigan Medical School, Ann Arbor, MI 48109 USA; 5 Center for Computational Medicine and Bioinformatics, University of Michigan Medical School, Ann Arbor, MI 48109 USA; 6 Comprehensive Cancer Center, University of Michigan Medical School, Ann Arbor, MI 48109 USA

## Abstract

**Background:**

Pathogenic *Escherichia coli* infections cause various diseases in humans and many animal species. However, with extensive *E. coli* vaccine research, we are still unable to fully protect ourselves against *E. coli* infections. To more rational development of effective and safe *E. coli* vaccine, it is important to better understand *E. coli* vaccine-associated gene interaction networks.

**Methods:**

In this study, we first extended the Vaccine Ontology (VO) to semantically represent various *E. coli* vaccines and genes used in the vaccine development. We also normalized *E. coli* gene names compiled from the annotations of various *E. coli* strains using a pan-genome-based annotation strategy. The Interaction Network Ontology (INO) includes a hierarchy of various interaction-related keywords useful for literature mining. Using VO, INO, and normalized *E. coli* gene names, we applied an ontology-based SciMiner literature mining strategy to mine all PubMed abstracts and retrieve *E. coli* vaccine-associated *E. coli* gene interactions. Four centrality metrics (i.e., degree, eigenvector, closeness, and betweenness) were calculated for identifying highly ranked genes and interaction types.

**Results:**

Using vaccine-related PubMed abstracts, our study identified 11,350 sentences that contain 88 unique INO interactions types and 1,781 unique *E. coli* genes. Each sentence contained at least one interaction type and two unique *E. coli* genes. An *E. coli* gene interaction network of genes and INO interaction types was created. From this big network, a sub-network consisting of 5 *E. coli* vaccine genes, including *carA*, *carB*, *fimH*, *fepA*, and *vat*, and 62 other *E. coli* genes, and 25 INO interaction types was identified. While many interaction types represent direct interactions between two indicated genes, our study has also shown that many of these retrieved interaction types are indirect in that the two genes participated in the specified interaction process in a required but indirect process. Our centrality analysis of these gene interaction networks identified top ranked *E. coli* genes and 6 INO interaction types (e.g., regulation and gene expression).

**Conclusions:**

Vaccine-related *E. coli* gene-gene interaction network was constructed using ontology-based literature mining strategy, which identified important *E. coli* vaccine genes and their interactions with other genes through specific interaction types.

**Electronic supplementary material:**

The online version of this article (doi:10.1186/s13326-017-0122-4) contains supplementary material, which is available to authorized users.

## Background

In addition to be harmless commensal strains, the versatile *E. coli* bacterial species includes many pathogenic variants [1]. Depending on the site of infection, pathogenic *E. coli* strains are divided into intestinal pathogenic *E. coli* (IPEC) and extraintestinal pathogenic *E. coli* (ExPEC). Example IPEC pathotypes include enteroaggregative *E. coli* (EAEC), enterohaemorrhagic *E. coli* (EHEC), enteropathogenic *E. coli* (EPEC), and enterotoxigenic *E. coli* (ETEC). The most common ExPEC pathotypes include uropathogenic *E. coli* (UPEC), meningitis-associated *E. coli* (MNEC), and avian pathogenic *E. coli* (APEC) [2]. These virulent *E. coli* strains cause various diseases (e.g., gastroenteritis and urinary tract infections) with big damages worldwide. For example, ETEC is estimated to cause 300,000 to 500,000 deaths per year, mostly in young children [3].

To prevent diseases caused by pathogenic *E. coli* infections, extensive vaccine research has been conducted [4–7]. The Vaccine Investigation and Online Information Network (VIOLIN; http://www.violinet.org/) [8, 9], a comprehensive web-based central resource for integrating vaccine research data curation and literature mining analysis, currently includes over 40 manually annotated *E. coli* vaccines. Among these vaccines, Dukoral, originally intended for protection against *Vibrio cholerae,* provides a moderate protection against ETEC infections in human [10]. However, there is no other licensed human *E. coli* vaccine available on the market, putting humans at risk of *E. coli* infections. Therefore, more active research is needed to develop new *E. coli* vaccines.

For rational pathogenic *E. coli* vaccine design, it is critical to understand *E. coli* gene functions and *E. coli*-host interaction mechanisms. With over 35,000 *E. coli*-related articles published in PubMed, it is impossible to read all these articles manually. Therefore, literature mining becomes critical. In addition to pathogenic strains, many *E. coli* strains are nonpathogenic. *E. coli* is also widely used as a model organism in microbiology studies and as a commonly used tool in recombinant biological engineering and industrial microbiology. Given so many *E. coli* strains and different *E. coli* usages, it has been a challenge in mining vaccine-related *E. coli* gene interactions from the large pool of literature reports. In this study, we use the commonly applied GENETAG-style named entity annotation [11], where a gene interaction can involve genes or gene products such as proteins. While human gene names are well normalized based on the HUGO Gene Nomenclature Committee (HGNC; http://www.genenames.org/), a similar gene nomenclature strategy for bacterial gene names has not been formed. However, it is possible to normalize bacterial gene names using the strategy of pan-genome. Specifically, a bacterial species can be described by its pan-genome, which is composed of core genes present in all strains, and dispensable (or accessory) genes present in two or more strains or unique to single strain [12, 13]. After a pan-genome is generated, the gene/protein names of the pan-genome of a bacterial species can be obtained by gene/protein name merging and cleanup from the annotations of all strains belonging to the bacteria species.

Integration of biomedical ontology with literature mining can significantly improve its performance. An ontology is a human- and computer-interpretable set of terms and relations that represent entities in a specific biomedical domain and how they relate to each other. Previously, we applied the community-based Vaccine Ontology (VO) [14] to enhance our literature mining of interferon-gamma related [15], *Brucella*-related [16], and fever-related [17] gene interaction networks within the context of vaccines and vaccinations. Recently, we have developed the Interaction Network Ontology (INO) and successfully applied it to the studies of vaccine gene interactions [18] and host-*Brucella* gene interactions [19]. In these studies, we used and expanded SciMiner [20], a natural language processing and literature mining program with a focus on scientific article mining. SciMiner uses both dictionary- and rule-based strategies for literature mining [20].

To better study gene interaction networks, we have also developed a literature mining strategy CONDL, standing for Centrality and Ontology-based Network Discovery using Literature data [17]. The centrality analysis here refers to the application of different centrality measures to calculate the most important genes (i.e., hub genes) of the resulting gene-gene interaction network out of biomedical literature mining. Four types of centrality measures have been studied: degree, eigenvector, closeness, and betweenness [17, 21]. The CONDL strategy was applied to extract and analyze IFN-γ and vaccine-related gene interaction network [21] and vaccine and fever-related gene interaction network [17], and our results showed that the centrality analyses could identify important genes and raise novel hypotheses based on literature mined gene interaction networks. In this study, we applied this approach, together with the pan-genome *E. coli* gene collection, to *E. coli* gene interaction networks using VO and INO to identify the crucial *E. coli* genes and interaction types.

## Methods

### Pan-genome based *E. coli* gene name normalization

*E. coli* gene names from *E. coli* K12 genome have been collected in EcoGene (http://www.ecogene.org/) [22], which were used as the basis for our *E. coli* gene name normalization. To integrate *E. coli* gene names from different *E. coli* genome annotations, we applied the pan-genome strategy [12, 13]. Specifically, out of 75 *E. coli* strains, we used the Vaxign program [23], which includes the OrthoMCL ortholog searching program [24], to generate an *E. coli* pan-genome that includes core *E. coli* genes shared by all strains, and dispensable genes present in two or more strains or unique to single strain. After the *E. coli* pan-genome was generated, the gene names of the pan-genome were reannotated by merging together different gene names from these *E. coli* strains when these gene names belong to the same genes of the pan-genome. The reannotated gene names were then used for next step literature mining.

### VO modeling of *E. coli* vaccines and genes used in *E. coli* vaccine development

*E. coli* VO ontology terms were obtained from the VIOLIN vaccines website (http://www.violinet.org/vaxquery/vaccine_query_process.php?c_pathogen_id[]=25) that contained 44 manually annotated *E. coli* vaccines. In addition to specific *E. coli* vaccine representations (terms), we also modeled and represented *E. coli* ‘*vaccine genes*’. Here, a ‘*vaccine gene*’ is defined as a microbial gene that has been used as a gene targeted or genetically engineered in at least one experimentally verified vaccine. For example, a vaccine gene may encode for a protective protein antigen, which can be expressed, purified, and used as the vaccine antigen component in a subunit vaccine. Some vaccine genes encode for virulence factors, and their mutations result in the generation of live attenuated vaccines [25].

#### VO/INO-SciMiner tagging of genes/interaction terms and vaccine terms

Our current study relies on the use of SciMiner (and its variant VO-SciMiner). The original SciMiner achieved 87% recall, 71% precision and 76% F-measure on BioCreAtIvE II Gene Normalization Task data [20]. In terms of identifying vaccine ontology terms, VO-SciMiner demonstrated 91% recall and 99% precision in the domain of *Brucella* vaccines [16]. In the current study, VO-SciMiner was further modified to be able to handle the compiled pan-genome-based *E. coli* genes with a more stringent name identification matching strategy.

**Fig. 1:**
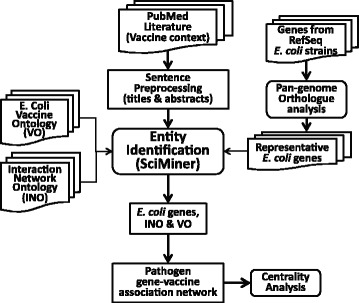
Project workflow. The presented study was limited to the literature in the vaccine domain. Representative *E. coli* genes, obtained through a pan-genome orthologue analysis, host genes as well as two established biomedical ontologies of interactions (INO) and vaccines (VO) were identified from the literature by SciMiner. Based on the co-occurrence among these identified entities, vaccine-associated *E. coli* gene-gene interaction network was generated and further analyzed to identify the central genes and enriched biological functions in this network

The abstracts and titles of all PubMed records published by the end of 2014 were used for the present literature mining study. Figure 1 illustrates our overall workflow. SciMiner [20] and its variations, specialized for specific ontologies (INO-SciMiner [18] and VO-SciMiner [16]) were used to process sentences from PubMed literature and to identify entities (*E. coli* VO terms, and INO terms). VO-SciMiner was modified to be able to handle the compiled pan-genome-based *E. coli* gene. In order to focus on the genes related to *E. coli* vaccine, the analysis was limited to the entities identified from the articles in *E. coli* and vaccine context, defined by a PubMed search of “*Escherichia coli* [MeSH]” and “vaccines [MeSH]”. Figure 1 illustrates the overall workflow of our approach.

### Co-occurrence analysis

The tagged genes were used to study the co-occurrence of genes and vaccines in the same sentences. First, an *E. coli* gene-gene interaction network was generated based on the sentence-level co-occurrence of *E. coli* genes. The *E. coli* gene-gene interactions were defined for any possible pairs of *E. coli* genes, two or more of which were identified from same sentence. The VIOLIN vaccine database [8, 9] includes 25 *E. coli* vaccine genes as shown on the VIOLIN website: http://www.violinet.org/vaxquery/query_detail.php?c_pathogen_id=25. These vaccine genes have also been represented in the VO. These *E. coli* vaccine genes were used in our ontology-based literature mining study, which aims to identify other *E. coli* genes that co-occur with these vaccine genes in the same sentences from peer-reviewed article abstracts.

This *E. coli* gene-gene interaction network was extended by INO to create a comprehensive vaccine-centered *E. coli* gene-gene interaction network. In this study, these additional entities were limited only to those in the same sentences, where two or more *E. coli* genes were mentioned.

### Centrality analysis

The collected gene-interaction networks were subject to centrality analysis. Four different centrality metrics were computed to identify the most important nodes (i.e., genes, vaccine genes, and INO terms) in the created interaction networks using the Cytoscape plug-in CentiScaPe [26]. The degree centrality of a node is the number of nodes that are its first neighbors (i.e., directly connected to the given node). The more connections a node has, the more central it is based on degree centrality. In degree centrality, all neighbors contribute equally to the importance of a node. In eigenvector centrality, a node contributes to the centrality of another node proportionally to its own centrality. A node is more central, if it is connected to many central nodes. The well-known PageRank algorithm for ranking web pages is also based on eigenvector centrality. Closeness and betweenness centralities depend on the position of a node in the network. Closeness centrality is based on the distance of a node to the other nodes in the network. The closer a node is to the other nodes, the more important it is considered to be. Betweenness centrality is based on the number of shortest paths connecting two nodes that pass over the given node. A node is more central, if it acts like a bridge in the network, i.e., lies on many shortest paths.

### Ontology-based hierarchical classification of interaction terms

All the interaction keywords identified in our literature mining were mapped to INO terms. The OntoFox tool [27] was used to extract these INO terms and additional terms related to these INO terms. The Protégé OWL editor [28] was used to visualize the hierarchical structure of these extracted terms.

## Results

### Pan-genome-based *E. coli* gene name normalization

Although EcoGene provides very good *E. coli* gene name annotations, it mainly covers the *E. coli* strain K12. However, many other *E. coli* strains are available and *E. coli* gene names are very complicated with different names across various strains. For example, the gene names “*iroN*” and “*fepA*” are synonyms, and *E. coli iroN* encodes for an outer membrane receptor FepA (http://www.ncbi.nlm.nih.gov/gene/7324526). Similarly, *E. coli* strain CFT073 gene C0393 (hemoglobin protease) has 100% sequence identity with the vacuolating autotransporter toxin (*vat*) gene from many other *E. coli* strains such as strain PAB48 (GenBank Accession ID: KR094946.1). Another example is the *E. coli* gene *rfaJ,* which has several synonyms such as *waaJ* (http://ecoliwiki.net/colipedia/index.php/rfaJ:Quickview). Such synonym information is often not reported in EcoGene. Therefore, we applied the pan-genome based strategy as detailed in the Methods section in order to get a more complete set of normalized *E. coli* gene names.

### VO modeling of vaccines and related vaccine genes

**Fig. 2:**
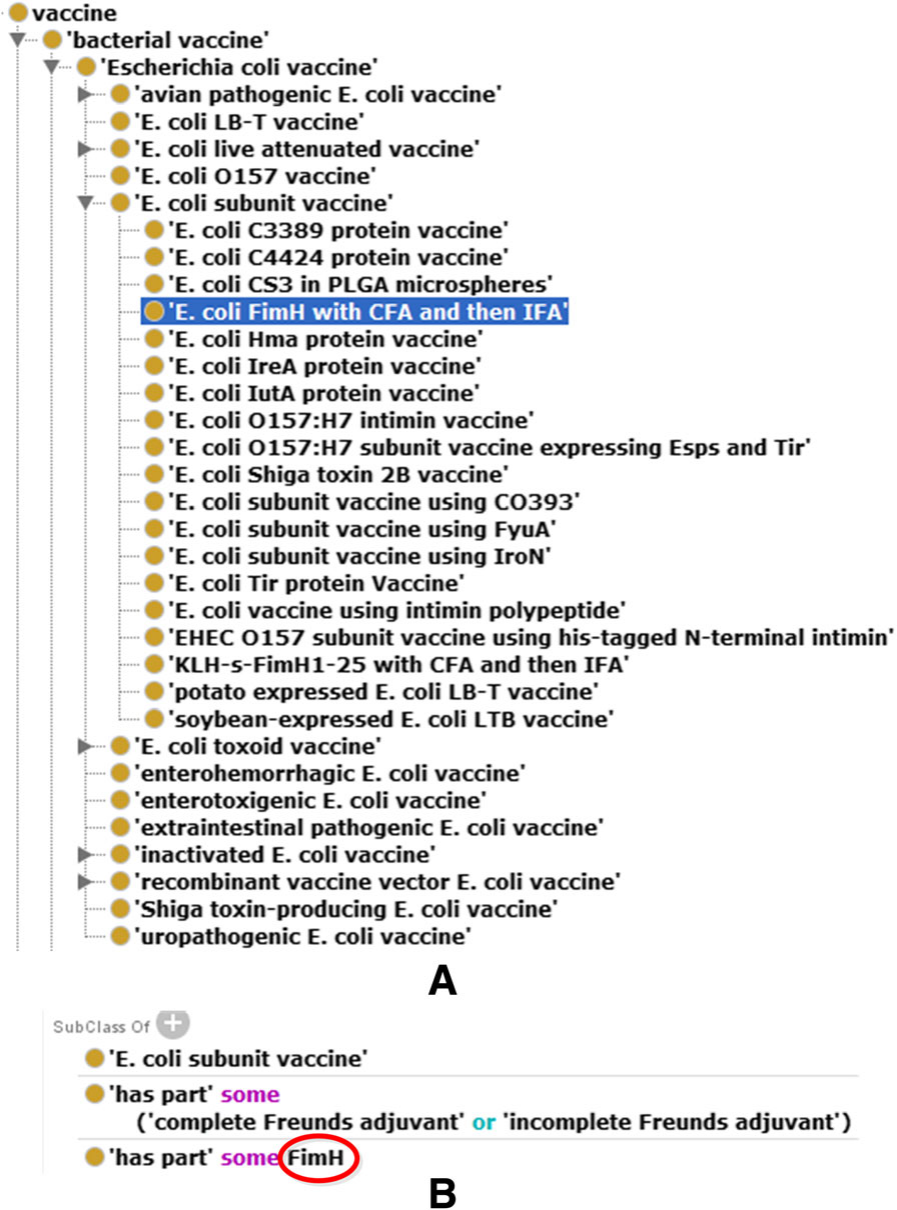
VO hierarchical structure and axioms of *E. coli* vaccines. **a** Vaccine hierarchy that shows the *E. coli* vaccines. **b** Axioms of the *E. coli* vaccine ‘*E. coli* FimH with CFA and then IFA’ (VO_0001168). The circled term ‘FimH’ is the *E. coli* protein FimH. These are screenshots with the Protégé OWL editor

The newest VIOLIN vaccine database includes 44 *E. coli* vaccines. Only approximately half of these vaccines existed in the initial release of VO back in 2012. In this study, we updated VO by including all these vaccines in VO, and we also added intermediate layer terms to better represent and organize the relations among these terms. VO also represents 25 *E. coli* vaccine genes and how these vaccine genes are used in *E. coli* vaccine formulations. Figure 2 provides an example of *E. coli* subunit vaccine ‘*E. coli* FimH with CFA and then IFA’. A subunit vaccine uses a subunit (typically a protein) of a pathogen organism as vaccine antigen. This vaccine uses the *E. coli* protein FimH (an *E. coli* fimbrial subunit and D-mannose specific adhesin) as the protective vaccine antigen, and it uses the complete Freund’s adjuvant (CFA) in the first vaccination and the incomplete Freund’s adjuvant (IFA) in the boost vaccination [29].

Some *E. coli* vaccines are live attenuated vaccines. One method to make a live attenuated vaccine is to knock out a virulence factor gene(s) in a wild-type virulent strain to make it less virulent (i.e., attenuated) but keep the antigenicity. For example, the *carA* and *carB* genes, which form a *carAB* operon, are virulent *E. coli* genes. Their mutations in an *E. coli* strain led the development of the mutant vaccine “*E. coli* carAB mutant vaccine” [30]. Such a virulence factor gene whose mutation leads to the generation of an experimental verified vaccine is named “virmugen” [25]. In VO, an ontological axiom is used to represent the relation between the vaccine and the mutated genes:

‘*E. coli* carAB mutant vaccine’: *not has_part some (carA or carB)*

In this ontological axiom, the relation ‘*not has part*’ means that the mutant vaccine strain does not have *carA* and *carB* genes in the mutated bacterial genome.

The VO representation of the vaccine-gene relations provides rationale for us to identify specific “vaccine genes” and study how these vaccine genes are related to other *E. coli* genes.

### Literature mining statistics and interaction network

The complete abstracts and titles from PubMed, published before December 31, 2014, were processed by SciMiner to identify *E. coli* genes, INO and VO terms. SciMiner identified 2,037 *E. coli* genes from 53,925 sentences in articles indexed with “*Escherichia coli* [MeSH]”. The study was further limited to the articles in the vaccine context (defined by ‘vaccines [MeSH]’), where SciMiner identified a total of 1,781 unique *E. coli* genes that were co-cited with at least one other *E. coli* genes at the sentence level. A total of 16,887 INO terms (mapped to 88 unique INOs) were also identified in 11,350 sentences.

**Fig. 3:**
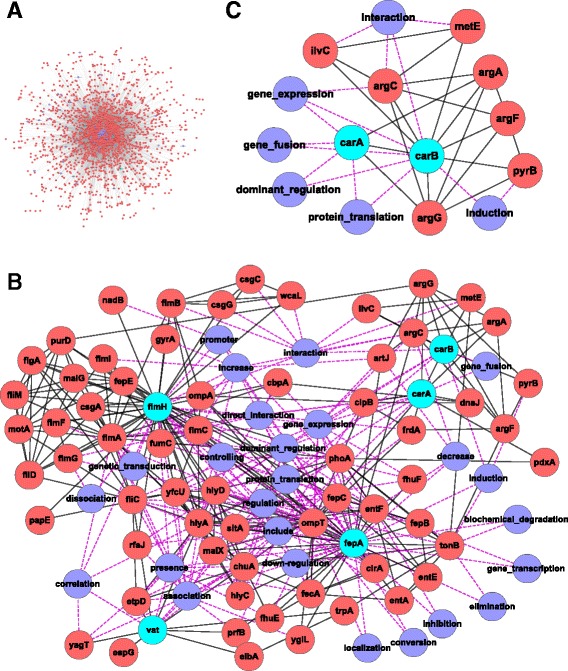
The interaction network among *E coli* genes and INO terms. **a** Interaction network among all *E. coli* genes co-cited at a sentence-level with INO terms in the vaccine context. **b** a sub-network focused on five *E. coli* genes (in *cyan* nodes) that are known to be used in *E. coli* vaccines. **c** a sub-network of two vaccine genes, *carA* and *carB*, and their immediate neighbors in (**b**). Gene names with additional synonyms were represented with the sign “|”. For example, “iroN|fepA” represents that this gene has two gene symbols “*iroN*” and “*fepA*”. Nodes in *red* represent *E. coli* genes, except cyan nodes, and nodes in *purple* are INO terms identified in the same sentences of these *E. coli* genes. The *pink dashed lines* represent interaction between *E. coli* gene and INO terms, while the *black solid lines* represent the interaction between *E. coli* genes

An interaction network of these *E. coli* genes and INO terms within the vaccine context was visualized in Fig. 3a. A subnetwork focused on known genes used in *E. coli* vaccines was generated as illustrated in Fig. 3b, which include 5 vaccine-genes (nodes in cyan), 62 *E. coli* non-vaccine genes (nodes in red), and 25 INO terms (nodes in purple).

As seen in the *carA* and *carB* sub-network (Fig. 3c), *carA* and *carB* were found in our literature mining to interact with each other through different interaction types including gene expression, gene fusion, dominant regulation, and protein translation. For example, the retrieved sentence corresponding to the gene fusion interaction (INO_0000106) between these two genes is:

“A construct was made in which the intergenic region between the contiguous *carA* and *carB* genes was deleted and the sequences encoding the carbamyl-phosphate synthetase subunits were fused in frame” [31].

In this case, after deletion of the intergenic region between these two genes, a fused *carA*-*carB* gene formed, and the resulting fusion protein was activated 10-fold relative to the native protein [31].

Meanwhile, our literature mining also found that *carA* or *carB* interacts with other genes. For example, *carB* interacts with *pyrB* through the induction interaction type (INO_0000122) as shown in the following sentence:

“In addition, however, exogenous uracil triggers cellulose production, particularly in strains defective in either *carB* or *pyrB* genes, which encode enzymes catalyzing the first steps of de novo UMP biosynthesis.” [32].

This sentence represents a complex interaction process. Specifically, the direct induction interaction is that exogenous uracil triggers cellulose production, and such interaction occurs when the *carB* or *pyrB* gene was defective. In this case, *carB* and *pyrB* genes are related, since both encode enzymes that catalyze the frist steps of de novo UMP biosynthesis [32]. In this case, the two genes do not directly interact through the induction type, i.e., it is not that *carB* (or *pyrB*) triggers *pyrB* (or *carB*). Instead, the two genes are involved in providing a condition to another induction interaction. Our study found that such cases occur frequently.

Other sub-networks centered on the other vaccine genes are available in Additional file 1. A Cytoscape file containing the *E. coli* gene-vaccine interaction network as well as the sub-networks centered on each vaccine-gene is available in Additional file 2.

### Centrality analysis

Our centrality analysis using the Fig 3b subnetwork identified the centralities of three types of nodes (*E. coli* vaccine genes, other *E. coli* genes, and INO terms) in the literature mined network as shown in Fig. 3b. By identifying top 10 nodes based on either of the four types of centrality scores, 19 central nodes were identified (Table 1). Out of the 19 “central” nodes, all the 5 *E. coli* vaccine genes are in the list. The result is reasonable since all the genes in Fig. 3b subnetwork are expected to interact with at least one of these five *E. coli* genes. Eight other *E. coli* genes are also found central in the list.

**Table 1:** The most central nodes in the network. The top 10 nodes based on Degree (D), Eigenvector (E), Closeness (C), and Betweenness (B) centrality metrics. The minimum (i.e., top) rank of each node based on any of the four centrality metrics is shown in the Min column

Type	Name	D	E	C	B	Min
Vaccine gene	*fimH*	1	1	2	1	1
Vaccine gene	*fepA*	2	2	1	2	1
*E. coli*	*fimA*	3	7	9	6	3
*E. coli*	*ompT*	4	4	3	4	3
*E. coli*	*hlyA*	5	3	4	-	3
INO	Inclusion	6	5	3	7	3
Vaccine gene	*vat*	-	-	-	3	3
Vaccine gene	*carA*	-	-	-	5	5
INO	protein translation	-	-	5	-	5
*E. coli*	*yfcU*	8	6	-	-	6
INO	gene expression	9	-	6	10	6
*E. coli*	*entF*	7	-	-	-	7
*E. coli*	*chuA*	9	8	7	-	7
*E. coli*	*tonB*	-	-	-	8	8
INO	dominant regulation	-	-	8	-	8
INO	association	9	9	9	-	9
INO	regulation	9	-	9	-	9
Vaccine gene	*carB*	-	-	-	9	9
*E. coli*	*hlyD*	-	10	-	-	10

The rankings of the terms are shown. Terms with the same centrality scores have the same ranking. Abbreviations here: “*E. coli*” - *E. coli* gene, “Vaccine gene” - *E. coli* vaccine gene; “INO” – INO term

Besides identifying the central *E. coli* genes, we also targeted the identification of central types of interactions among these genes in the created vaccine associated *E. coli* gene interaction network. Therefore, INO terms (interaction types) were represented as nodes in the network. Six INO terms were identified in the top node list (Table 1). These terms (e.g., gene expression and regulation) represent the most commonly identified interaction types in vaccine-related *E. coli* gene interaction studies.

Different centrality measures provide different aspects of the network (Table 1), since they define centrality in different ways and capture central nodes based on different aspects. While some node are central based on all four centrality metrics, some are identified as central by only one or two of the centrality metrics. Overall, degree centrality and eigenvector centrality results are similar. Interestingly, three out of the five vaccine genes were ranked in the top 10 only by the betweenness centrality metric, suggesting that these three vaccine genes are critical to link together different sections in the network. A node may be considered as important, even if it is identified as central based on only one centrality metric. Therefore, to summarize the importance of a node, the minimum (i.e.*,* top) rank of each node based on any of the four centrality metrics is shown in Table 1.

### INO ontology-based analysis of interaction types

Here is one example sentence identified from our study: “Complementation experiments indicated that both the major fimbrial subunit gene, *fimA*, and the *fimH* gene in combination with either the *fimF* or the *fimG* gene were required for mannose-specific adhesion.” [33].

This sentence represents the INO interaction type ‘regulation’ (INO_0000157). Specifically, the four genes *fimA, fimH, and fimF (or fimG*) were found to regulate (“were required for”) the mannose-specific adhesin [33]. Note that in our literature mining, the regulation relation does not have to be one gene regulating another gene; it is also allowable for both genes regulating for a specific phenotype.

For the INO interaction type detection, we used the literature mining keywords collected in the INO. Specifically, in INO, we used the annotation property ‘has literature mining keywords’ (INO_0000006) to assign many keywords used to represent the interaction type. For example, “required” is a keyword assigned for the INO interaction type ‘regulation’.

**Fig. 4:**
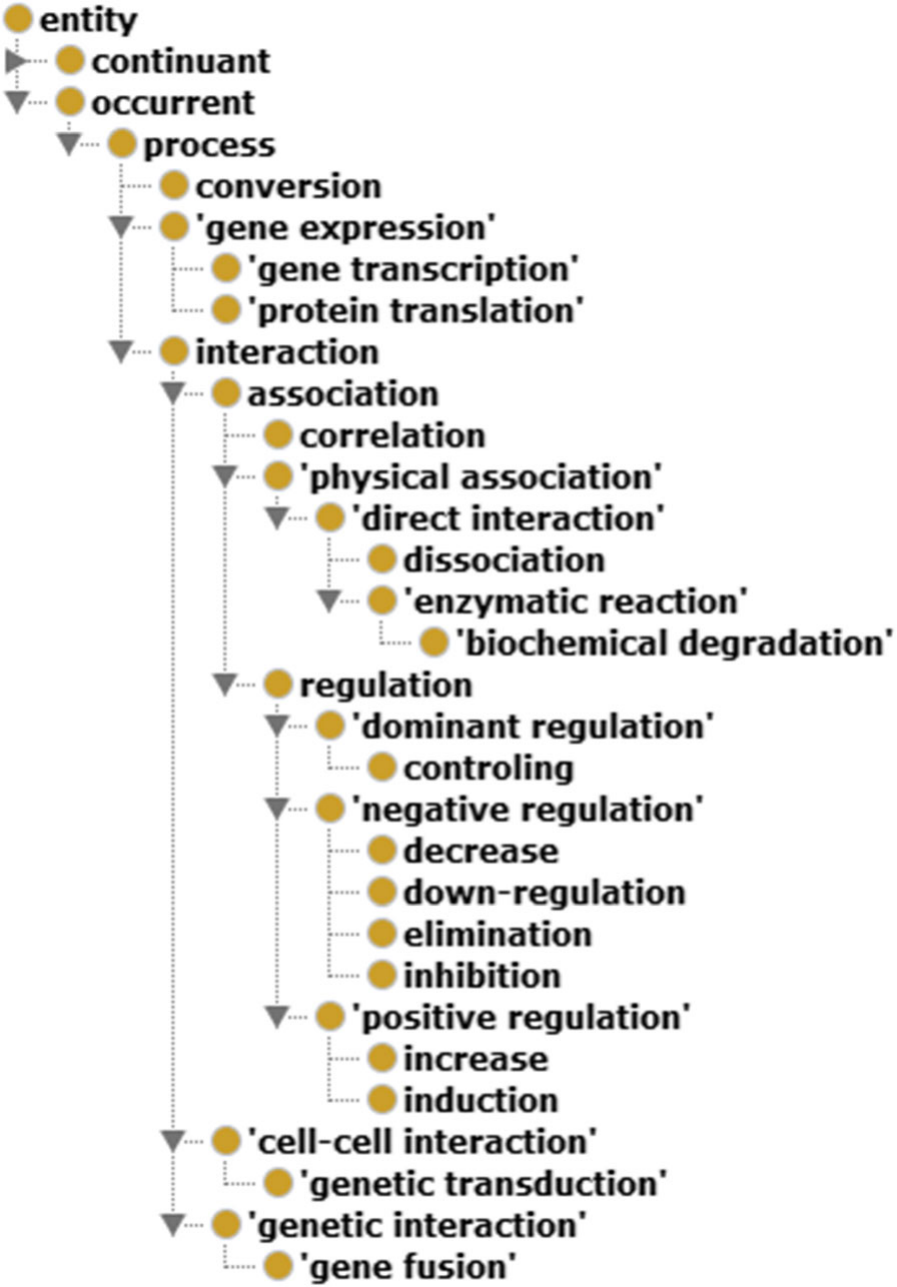
INO hierarchy of 25 interaction keywords identified in the vaccine-related *E. coli* gene interaction network. OntoFox [27] was used to extract the hierarchical structure among the 25 identified INO types. The OntoFox option of “includeAllIntermediates” was used in the process. The Protégé OWL editor was used for structure visualization

From our literature mining study, 25 specific INO interaction types were identified. The hierarchical structure of these 25 INO interactions types is shown in Fig. 4. As shown in this figure, the most common interaction type is various types of ‘regulation’, including positive, negative, and dominant regulation types. Other interaction types such as direct physical interactions and gene expression types (including transcription and translations) are also included. Such an INO hierarchical analysis clearly illustrates how different genes interacted with each other based on the reported literature papers.

## Discussion

The contributions of this study are multiple. First, this study for the first time applied ontology-based literature mining method to analyze vaccine-related *E. coli* gene interaction network using all PubMed abstracts. Considering the status of *E. coli* in microbiology, infectious diseases, and the whole biology, such a study is important. Second, our study employed pan-genome-based approach to normalize *E. coli* gene names across various strains. Third, this study represents the first-time application of applying both VO and INO in supporting literature mining of pathogen and vaccine-related gene-gene interactions. Fourth, we further demonstrated that the centrality-based analysis enhanced our ability in identifying hub or critical genes or nodes in the *E. coli* gene-vaccine intearction network.

The identification of those other *E. coli* genes that interact with known *E. coli* vaccine genes from our study provides scientific insights on *E. coli* vaccine research and development. These genes as a whole provide an explanation on the functions and biological processes of these genes preferred for vaccine development. These genes also provide new candidates for future vaccine development. It should be noted that not all *E. coli* vaccine genes were identified in our literature mining process, since our analysis focuses on retrieving gene-gene interactions instead of individual genes.

Compared to our previous vaccine-related *Brucella* gene interaction literature mining study [16], the current study includes the more challenging *E. coli* species and also for the first time employed a new INO-based interaction type analysis approach. In general, our study found many commonly reported interaction types (e.g., expression and regulation) from the *E. coli* vaccine-gene interaction network. We also found that different types of regulation often are not about the direct regulatory interactions between two genes (e.g. gene A regulates gene B). Instead, they are often related to regulatory interactions between the genes and another interaction process or phenotype. For example, as shown in the “mannose-specific adhesion” sentence described in the Results section, the gene *fimA* and the gene *fimH* were both required for a phenotype: mannose-specific adhesion [33], rather than they had a direct interaction. Another example is the *carB* vs *pyrB* interaction, which was also shown in the Results section, where the two genes participate in a pathway and a defective pathway process results in the occurrence of an induction interaction [32]. These two examples represent quite complex interactions that involve multiple components and relationships that are represented by multiple literature keywords as shown in our previous studies [18, 34]. Further research is required to automatically identify such specific and complex patterns from the biomedical literature.

It is possible that tagged *E. coli* genes from our literature mining and their associated ortholog genes in other bacteria may likely co-occur with most vaccines for various bacteria (instead of only *E. coli*). This aspect of study is out of our scope for this study since we only focus on *E. coli* in this study. However, our previous INO-based study found that many genes co-occur in sentences with vaccines, and we even developed an INO-based Fisher’s exact test to perform enrichment analysis of tagged genes in the scope of INO [18]. It is noted that the previous INO-based study focused on human genes [18] while our current study focuses on bacterial genes. However, we envision that bacterial genes would perform similarly. Our previous VO-based *Brucella* gene-vaccine interaction study identified many interesting patterns among the *Brucella* genes as well [16]. Furthermore, many studies have found that the collection of bacterial genes, proven to be useful in vaccine development, often share common characteristics [25, 35, 36]. For example, systematic analysis of a collection of experimentally verified protective bacterial genes revealed multiple conserved domains (or called motifs) and preferred subcellular localizations among protective antigens [35, 36]. The collection and analysis of a set of virulence factors (i.e., “virmugens”) whose mutations led to experimentally verified live attenuated vaccines also discovered many enriched virmugens patterns, for example, the frequent usage of bacterial *aroA* genes as virmugens, and virmugens often involving metabolism of nutrients (e.g., amino acids, carbohydrates, and nucleotides) and cell membrane formation [25]. These results out of systematical analyses facilitate rational vaccine design. More researches are warrantied to apply literature mining to identify more specific vaccine-associated gene/protein patterns and underlying biological and immunological mechanisms.

Our literature mining method identifies gene-gene interactions based on sentence-level co-citation analysis. The directionality of the extracted gene-gene interactions is not detected by the current SciMiner. Therefore, the generated gene-gene interaction network is undirected and the centrality scores are computed on this undirected network. For example, if a sentence states that Gene A activates Gene B, an undirected edge between Gene A and Gene B is included in the gene-gene interaction network. The information that the directionality of the interaction is from Gene A to Gene B is lost. In our future work, we will develop new text mining and statistical methods to identify the directionality information regarding gene-gene interactions. With the directionality of extracted gene-gene interactions, it would be easier to find “provider” or “consumer” roles for different genes. We will investigate how centrality analysis is affected when directionality information is incorporated. A direction-based importance metric, such as SimRank [37], can be measured to provide direction-based weights to network nodes and generate more interesting results.

Our future directions will be multiple. First, we plan to improve our pan-genome-based gene name normalization method to cover other pathogens and to include such a strategy automatically in our SciMiner pipeline to study other pathogens (including bacteria, viruses, and parasites). The performance of our SciMiner pipeline in host-pathogen interaction literature mining will be thoroughly evaluated using manually curated documents. Second, we also plan to apply our methods to study host-pathogen/vaccine interactions. In addition, we will extend the INO modeling to better support ontology-based literature mining. Furthermore, statistical and machine learning methods [38, 39] will be explored to improve our literature mining and downstream analysis.

## Conclusions

In this study, we first used a pan-genome-based approach to collect and normalize *E. coli* genes and corresponding gene names, relied on the Vaccine Ontology to obtain *E. coli* vaccines and vaccine genes, and applied the Interaction Network Ontology to obtain possible interaction keywords. These *E. coli* gene names, vaccine names, vaccine genes, and interaction keywords were then combinatorially used by SciMiner to process all PubMed abstracts to construct a vaccine-related *E. coli* gene-vaccine interaction network. From the contructed interaction nework, our centrality analysis further identified hub or critical *E. coli* genes and the types of the interactions involved in the network. New insights have been identified using our systematic analysis. To our knowledge, this is the first study of applying pan-genome and ontology-based literature mining strategy to construct *E. coli* gene interaction network and perform systematic centrality analysis.

## Additional files

Additional file 1: A PDF file containing three other gene-vaccine sub-networks. (DOCX 932 kb)
Additional file 2: A Cytoscape session file containing the *E. coli* gene-vaccine interaction network and its sub-networks. (CYS 76 kb)

## Abbreviations

APEC: Avian pathogenic E. coli
CONDL: Centrality and ontology-based network discovery using literature data
EAEC: Enteroaggregative E. coli
EHEC: Enterohaemorrhagic E. coli
ExPEC: Extraintestinal pathogenic E. coli
HGNC: HUGO gene nomenclature committee
INO: Interaction network ontology
IPEC: Intestinal pathogen E. coli
MNEC: Meningitis-Associated E. coli
UPEC: Uropathogenic E. coli
VIOLIN: Vaccine investigation and online information network
VO: Vaccine ontology
