# Supplementary material for: Ontology-based literature mining of E. coli vaccine-associated gene interaction networks
Source: J Biomed Semantics. 2017 Mar 14;8:12. doi: 10.1186/s13326-017-0122-4 (PMC5348867; doi:10.1186/s13326-017-0122-4)
Supplement: Additional file 1: — A PDF file containing three other gene-vaccine sub-networks. (DOCX 932 kb) [file 13326_2017_122_MOESM1_ESM.docx]

**Supplementary Figure 1. Sub-network of vaccine gene - *eaaA***


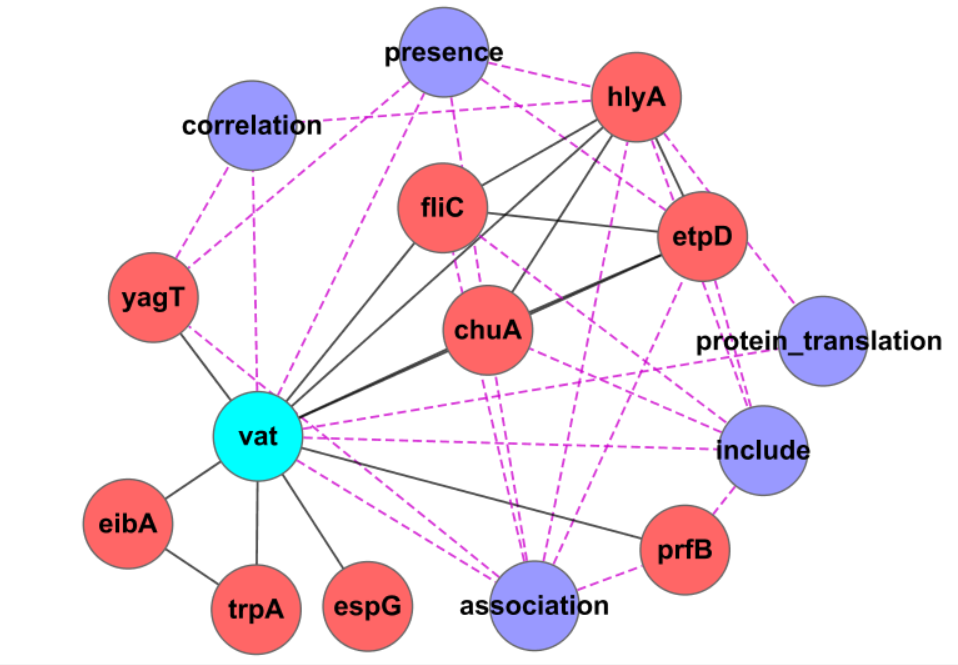


This is a sub-network of *eaaA* vaccine gene and its immediate neighbors in main Figure 3B. Nodes in red represent *E. coli* genes, except cyan nodes, and nodes in purple are INO terms identified in the same sentences of these *E. coli* genes. The pink dashed lines represent interaction between *E. coli* gene and INO terms, while the black solid lines represent the interaction between *E. coli* genes.

**Supplementary Figure 2. Sub-network of vaccine gene – *fimH***


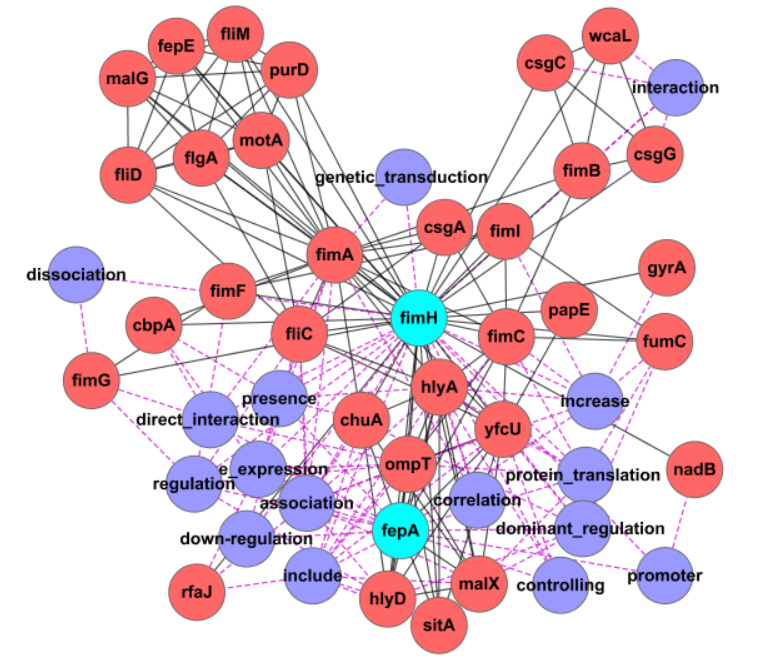


This is a sub-network of *fimH* vaccine gene and its immediate neighbors in main Figure 3B. Nodes in red represent *E. coli* genes, except cyan nodes, and nodes in purple are INO terms identified in the same sentences of these *E. coli* genes. The pink dashed lines represent interaction between *E. coli* gene and INO terms, while the black solid lines represent the interaction between *E. coli* genes.

**Supplementary Figure 3. Sub-network of vaccine gene – *iroN***


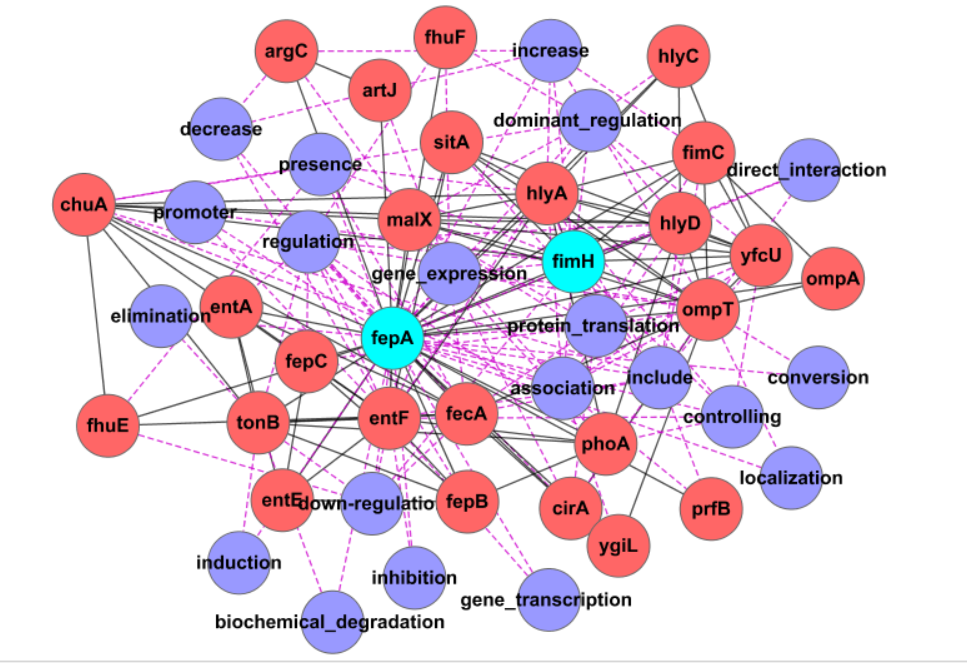


This is a sub-network of *iroN (fepA)* vaccine gene and its immediate neighbors in main Figure 3B. Nodes in red represent *E. coli* genes, except cyan nodes, and nodes in purple are INO terms identified in the same sentences of these *E. coli* genes. The pink dashed lines represent interaction between *E. coli* gene and INO terms, while the black solid lines represent the interaction between *E. coli* genes.
